# *Operando* Multi-modal Synchrotron Investigation for Structural and Chemical Evolution of Cupric Sulfide (CuS) Additive in Li-S battery

**DOI:** 10.1038/s41598-017-12738-0

**Published:** 2017-10-11

**Authors:** Ke Sun, Chonghang Zhao, Cheng-Hung Lin, Eli Stavitski, Garth J. Williams, Jianming Bai, Eric Dooryhee, Klaus Attenkofer, Juergen Thieme, Yu-chen Karen Chen-Wiegart, Hong Gan

**Affiliations:** 1 Sustainable Energy Technologies Department, Brookhaven National Laboratory, Upton, NY 11973 USA; 2 Department of Materials Science and Chemical Engineering, Stony Brook University, Stony Brook, NY 11794 USA; 3 National Synchrotron Light Source II, Brookhaven National Laboratory, Upton, NY 11973 USA

## Abstract

Conductive metal sulfides are promising multi-functional additives for future lithium-sulfur (Li-S) batteries. These can increase the sulfur cathode’s electrical conductivity to improve the battery’s power capability, as well as contribute to the overall cell-discharge capacity. This multi-functional electrode design showed initial promise; however, complicated interactions at the system level are accompanied by some detrimental side effects. The metal sulfide additives with a chemical conversion as the reaction mechanism, e.g., CuS and FeS_2_, can increase the theoretical capacity of the Li-S system. However, these additives may cause undesired parasitic reactions, such as the dissolution of the additive in the electrolyte. Studying such complex reactions presents a challenge because it requires experimental methods that can track the chemical and structural evolution of the system during an electrochemical process. To address the fundamental mechanisms in these systems, we employed an *operando* multimodal x-ray characterization approach to study the structural and chemical evolution of the metal sulfide—utilizing powder diffraction and fluorescence imaging to resolve the former and absorption spectroscopy the latter—during lithiation and de-lithiation of a Li-S battery with CuS as the multi-functional cathode additive. The resulting elucidation of the structural and chemical evolution of the system leads to a new description of the reaction mechanism.

## Introduction

The lithium-sulfur battery has been studied intensively as a next generation electrochemical energy storage device because of its superior theoretical energy density of 2600 Wh kg^−1^, which surpasses that of current state-of-the-art Li-ion batteries with energy density of 300–600 Wh kg^−1^, depending upon intercalation chemistries^1–4^. However, the nature of the chemical species and reactions in a functional Li-S battery lead to several critical challenges^3–5^. Two of the prominent issues are the high solubility of intermediate polysulfide species in the electrolyte and the poor electrical conductivity in the two end products—Li_2_S and sulfur—which result in poor cycling performance and low active-material utilization in prototype cells. It has been found that the trapping of sulfur in a myriad of porous and conductive carbon nanostructures by means of surface coating, encapsulation, and impregnation can help solve these two problems at one stroke, with greatly enhanced performance^6–11^. In order to make carbonaceous sulfur hosts effective, generally, at least 20–30 wt.% of carbon has to be incorporated into the sulfur cathode, which decreases the cell’s effective energy density. Therefore, a need exists to develop new sulfur hosts that can offer enhanced conduction and also react with lithium to offer extra capacity.

The concept of multi-functional electrode design provides a crucial path forward in energy storage and conversion fields, even beyond battery research^12–16^. Furthermore, this approach is able to create new electrode materials and architectural designs for innovative functions – such as devices that can provide high energy-density and power simultaneously^17^. In fact, some preliminary results have pointed to transition metal sulfides as potential candidates to act as multi-functional additives in Li-S batteries, such as TiS_2_^18,19^, MoS_2_^20^, CuS^21^, CoS_2_^22^ and FeS_2_^23^. Each of these compounds is both electrically conductive and contributes considerable capacity. By reacting with lithium in the voltage range of 2.6 V-1.0 V vs. Li/Li^+^, they are compatible with the operational voltage of a Li-S battery through either intercalation or conversion mechanisms. Indeed, they have been investigated individually as sulfur electrode additives and showed beneficial effects in capacity retention and high power performance.

CuS is a highly attractive choice due to its high conductivity (870 S cm^−1^) and the two voltage plateaus around 2.0 V and 1.7 V during lithiation that overlap with the majority of the sulfur electrode discharge. Moreover, these high voltage cut-offs allow the use of the lithium-anode-passivating additive LiNO_3_ in the electrolyte without the undesirable nitrate-anion reduction on the cathode, which happens at ~1.6 V vs. Li/Li^+^^24^. In addition, its theoretical energy density of 961 Wh kg^−1^, in full conversion into Li_2_S and Cu, can also compensate for its occupied weight and volume within the electrode. Our previous studies using CuS as a multi-functional additive showed an enhanced discharge power capability with improved sulfur utilization in Li-S batteries^25^. However, sulfur-CuS hybrid electrodes experienced Cu cation dissolution and deposition on lithium that destroys the anode’s solid-electrolyte interface (SEI) layer, which leads to cell failure in a few cycles. This observation represents a design challenge in multi-functional electrodes: while introducing new components with desirable properties, parasitic reactions may occur and hinder the original design intentions.

**Figure 1:**
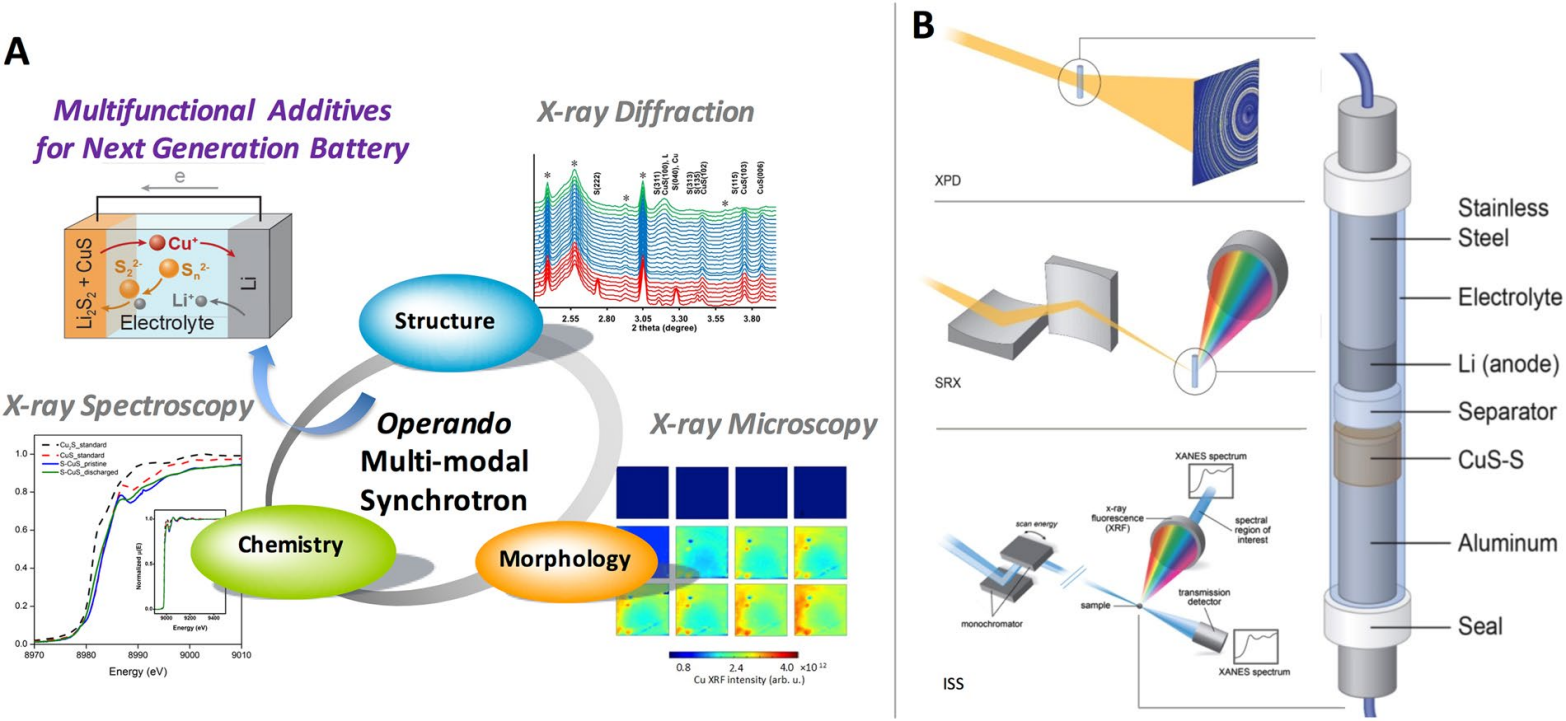
(**A**) Schematic to illustrate the concept of *operando* battery reaction with the multi-modal synchrotron approach. (**B**) Schematic of the experiment set-up and battery cell design for the synchrotron x-ray multi-modal study.

To address such challenges and to guide future electrode design, we need to reveal how various mechanisms drive structural, chemical, and morphological material evolution in complex systems. In this study, we apply a multi-modal *operando* synchrotron x-ray approach to study the interaction of CuS and polysulfide species to obtain insight into the dissolution mechanism of CuS when used as an additive in a sulfur electrode. In addition, CuS exhibits chemical conversion as its reaction mechanism against Li. Conversion-based additives can lead to higher theoretical capacity in energy storage systems; therefore, understanding the fundamental reaction mechanisms and limitations of these, such as the interaction of the S and the metal sulfide in the hybrid cell, is important. Here, we combine X-ray Powder Diffraction (XPD), X-ray Absorption Spectroscopy (XAS), and X-ray Fluorescence (XRF) microscopy to address the changes in material structure, chemistry, and elemental distribution. The concept of this multi-modal approach is summarized in Fig. 1A. We utilized XPD to study the phase transformation and long-range ordering in the crystalline phase, XAS to study the oxidation states and short-range ordering, and XRF microscopy to investigate the evolution of the elemental distribution. All measurements were conducted *operando*, as copper dissolution and re-deposition occur during cycling. Through this multimodal approach, we are able to further validate the hypothesis that one of the mobile polysulfide species generated by sulfur-component discharge is responsible for Cu^+^ dissolution from CuS or Cu_2_S^25^, while demonstrating an approach to support future multi-functional electrode developments.

## Results and Discussion

### Design feature of synchrotron multi-modal Operando battery cell

To enable our multi-modal approach, we first designed a cell that is fully compatible with all techniques and three different synchrotron x-ray beamlines at National Synchrotron Light Source (NSLS II) of Brookhaven National Laboratory. A schematic of the resulting *operando*-battery-cell design is shown in Fig. 1B. Importantly this design not only allows measurements to be conducted at the cathode and anode, but is optically transparent to enable in-line optical microscopy and alignment at the x-ray beamlines. These characteristics are critical to spatially resolve the reactions from different components and at multiple locations within the cell, one of our main research aims. The cell construction procedure (described in the experimental section) was optimized in an effort to ensure that the cell performance and electrochemical reaction profiles still closely represent the reactions observed in coin cells. Moreover, the versatile and simple design using economical parts allows many cells to be constructed for each synchrotron experiment, compared to other designs that require specialized components. While both approaches yielded success in conducting synchrotron experiments *operando*, our design allows us to adjust to the requirements of the beamlines effectively.

Differences in this cell design parameters must be compared with the conventional coin cell design. Our *operando* cell has a higher internal resistance due to the longer distance between the cathode and the anode (~1 mm vs. 50 μm). It also has a higher electrolyte to cathode weight ratio, which will impact the dissolution and migration of the cell-discharge soluble species. In addition, the relatively small electrode size may introduce uncertainty in accurate electrode weight measurements. Therefore, slow discharge rates are used in this study to ensure the reaction mechanisms remain representative.

### Chemical evolution by *operando* synchrotron x-ray diffraction

**Figure 2:**
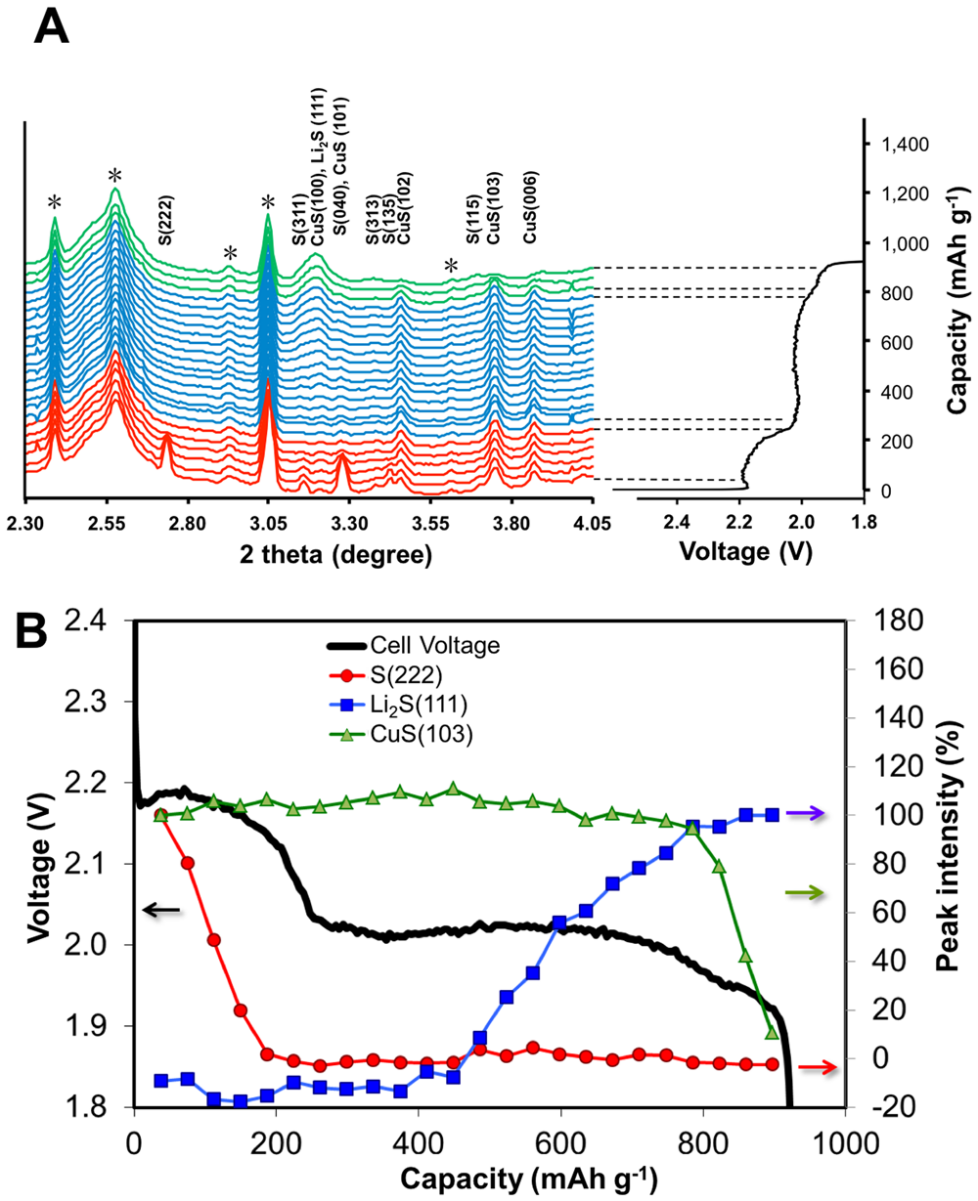
(**A**) *Operando* XPD patterns from the sulfur-CuS hybrid electrode aligned with its electrochemical capacity-voltage profile during lithiation; diffraction peaks from the cell construction components are labelled with “*”; (**B**) Integrated intensity of S (222), CuS (103), and Li_2_S (111) are plotted as a function of depth of discharge in comparison with the cell discharge voltage profile (solid black line).

The long-range structural evolution in the hybrid sulfur-CuS electrode during reaction was revealed by XPD. *In situ* X-ray diffraction has been demonstrated to be an effective tool in probing structural evolution during battery reactions, including Li-S batteries^26,27^ and other battery systems^28,29^. The X-ray powder diffraction patterns collected during the discharge of a sulfur-CuS hybrid electrode are overlaid in Fig. 2A; each individual pattern is correlated with a corresponding level of discharge in the voltage profile on the right. In the capacity-voltage profile, the two distinct regions in the discharge process can be clearly discerned. The sloped region above 2.0 V is ascribed to the generation of high-order polysulfides (Li_2_S_n_, 3 ≤ n ≤ 8) and the relatively flat-plateau region around 2.0 V is generally ascribed to the conversion of low-order polysulfides (Li_2_S_n_, n ≥ 3) into Li_2_S_2_ and finally Li_2_S deposition in the cathode^5^. Owing to the presence of CuS, a portion of the cell capacity should be from CuS in this voltage range. Assuming that CuS is reduced to Cu_2_S, the theoretical capacity contribution from CuS is 93 mAh g^−1^, normalized by the sulfur mass. The CuS discharge process (2Li + 2CuS =  > Cu_2_S + Li_2_S) generally happens at ~2.1 V at a low rate of ~C/10^25^, but the exact voltage level associated with CuS discharge cannot be directly predicted because sulfur’s plateau discharge overlaps with it. The end of the discharge capacity is calculated to be 924 mAh g^−1^ of sulfur, which is close to 55% sulfur utilization and consistent with the literature.

The Miller indices of assigned Bragg peaks are shown above the profiles in Fig. 2A. At the beginning of the discharge, only reflections from sulfur and CuS (Covellite) are present. In the following lithiation process the only new diffraction peak emerging is Li_2_S (111). The Li_2_S_2_ phase that is generally believed to develop during the early stage of plateau discharge region has not been detected ; this is in agreement with the *in situ* XPD study of a Li-S battery system reported elsewhere^30^. Lithiation of CuS is supposed to generate Cu_2_S in its first voltage plateau at ~2.14 V with slow rate, < C/10, by 2Li + 2CuS =  > Cu_2_S + Li_2_S and subsequently Cu metal in the second voltage plateau at ~1.78 V with slow rate, by 2Li + Cu_2_S =  > 2Cu + Li_2_S^31^. However, neither of them was detected during the *operando* XPD experiment here. It was shown in one of the *in situ* XRD studies of CuS under lithiation^32^ that the diffraction peaks of Cu_2_S (Chacolcite) generated by lithiation are much weaker than the starting CuS and end product Cu metal, which suggests that the Cu_2_S intermediate probably has a tendency to exist as highly amorphous domains under these conditions. This amorphous structure can explain why Cu_2_S was not identified in our diffraction data. The reason why Cu metal has not been detected here as well is that the lower cut-off voltage is not enough to initiate the second voltage plateau of the CuS discharge, which is generally around 1.7 V^16^. Additional *operando* XRD data from a full discharge-charge cycle can be found in Figure S1, where a gradual decrease of Li_2_S (111) was observed during the charging of the hybrid cell. No additional crystalline phase transformation was detected during the charging by *operando* XRD, indicating that some amorphous phases were present. This reduction in crystallinity will be further investigated and discussed in the XAS experimental results section.

To obtain a clear trend of the evolution of the three phases identified here as the discharge of sulfur-CuS hybrid electrode progresses, the peak area of three relatively strong reflections S (222), CuS (103), and Li_2_S (111) were chosen to be integrated at each depth of discharge and plotted together against discharge capacity in Fig. 2B. The other peaks not included in the analysis follow the same trend qualitatively. The S (222) peak area decreases monotonically right after the start of lithiation, and it drops to almost zero at 224 mAh g^−1^ of sulfur (~21% of total delivered capacity), which is right before the transition point between the sloped region and the plateau region. This is consistent from earlier report by Cañas *et al*.^30^. The rapid decrease of sulfur peak signal in the initial stage of discharge is caused by the conversion of crystalline sulfur into polysulfides that dissolve into the electrolyte. The complete disappearance of the S(222) peak also indicates that all sulfur content in the composite electrode took part in the reaction; this suggests that the generally poor efficiency of sulfur utilization in Li-S battery (<60%) is not because a great portion of sulfur is isolated and inaccessible from electrochemical reaction. It is probably due to the inefficiency in later processes, such as the materials loss caused by the redistribution of polysulfides in the cell. The transition point between the sloped discharge and the plateau discharge regions has been ascribed as the onset of Li_2_S_2_/Li_2_S nucleation and the combined evidence of the disappearance of the S(222) signal and this transition point indicates that the Li_2_S_2_/Li_2_S nucleation is not initiated until sulfur is fully consumed. The Li_2_S does not appear until 485mAh g^−1^, (43% of total delivered capacity), and its intensity increases steadily through the rest of discharge. Apparently there is a gap between the disappearance of ordered sulfur and the emergence of ordered Li_2_S. This might be because Li_2_S_2_ is the first to nucleate after the solid sulfur is fully converted into polysulfides and Li_2_S_2_ remains amorphous. What is most surprising in this study is that CuS signal does not decrease until the end of the discharge. Based on a report by Jache *et al*.^31^, CuS’s diffraction peaks are very sensitive to the depth of discharge and decrease in intensity as the lithiation process starts. The late decrease of the CuS diffraction signal indicates that CuS’s lithiation process happens at the very end of the discharge in this sulfur-CuS hybrid electrode cell. Therefore, it should remain in CuS phase before 747 mAh g^−1^ sulfur (85% of total delivered capacity). Indeed, the voltage of CuS’s first plateau discharge and sulfur’s plateau discharge region are very close to each other at a similar C-rate with the same cell designs^25^, both were measured at around 2.1 V in a coin cell at a low rate near equilibrium. It is thus very difficult to predict which one will experience lithiation earlier than the other. Combining the information from XPD and electrochemical discharge in our *operando* study, it has been determined that the polysulfide reduction to Li_2_S_2_ and Li_2_S and the subsequent conversion of Li_2_S_2_ to Li_2_S happen ahead of CuS reduction. This is evident by the two distinctive voltage plateaus at ~2.02 V followed by ~1.95 V. The observed discharge voltage plateau of CuS is lower than expected^25^ most likely due to the additional cell voltage polarization during the cell discharge, which may be induced via a higher cell internal resistance of the *operando* cell design. Nevertheless, the reaction mechanisms elucidated in this study are still representative of this hybrid cathode system.

### Structural and chemical evolution by *operando* synchrotron x-ray absorption spectroscopy

**Figure 3:**
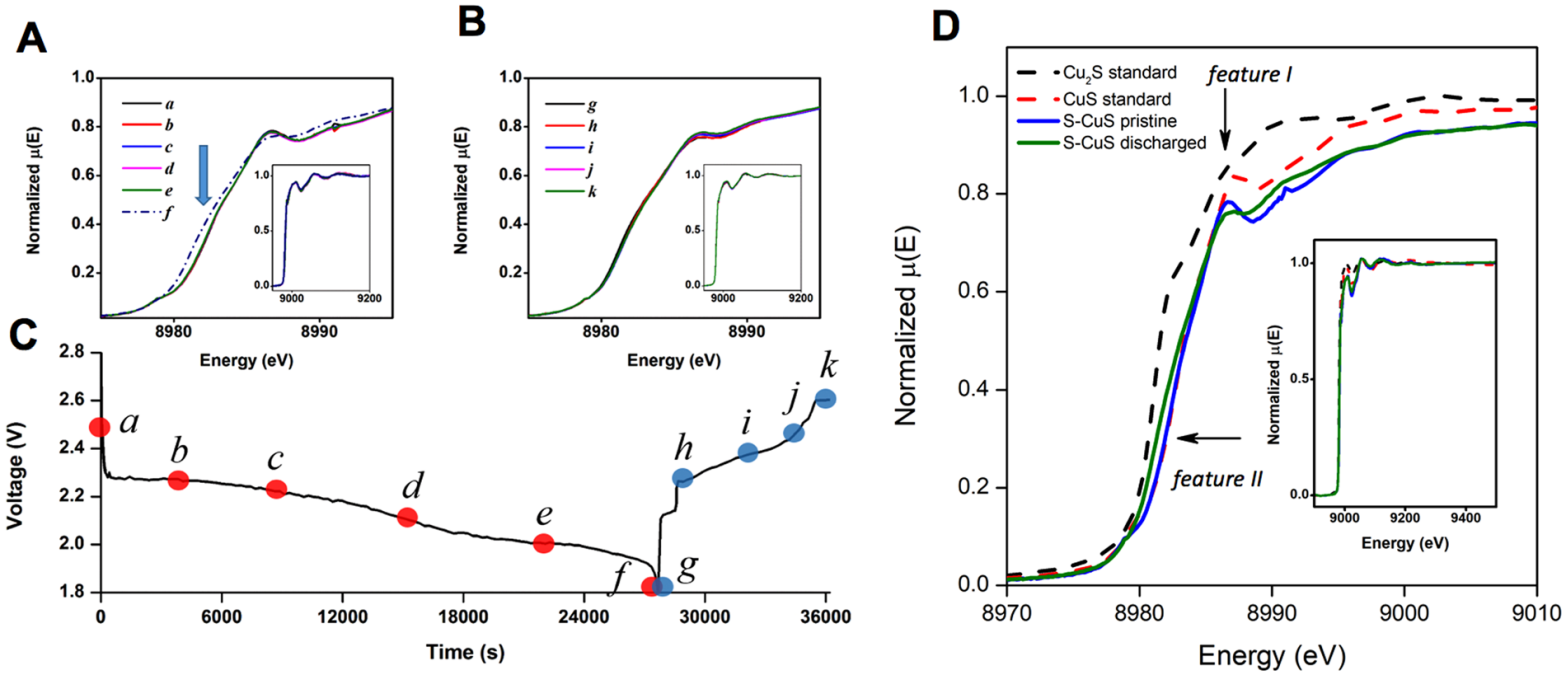
(**A**,**B**) Series of operando Cu K-edge XANES for discharge/charge reaction of sulfur-CuS hybrid electrode: curves a-f and g-k are corresponding to data points labelled in capacity-voltage profile in (**C**). The inserts in Figure **A**,**B** show the full EXAFS spectra. The arrow in Figure (**A)** indicates the location of the shoulder, corresponds to Cu_1+x_S phase. (**D**) Cu K-edge XANES spectra of CuS and Cu_2_S standards compared with pristine (curve a in figure (**A**)) and discharged (curve f in figure (**A**)) sulfur-CuS hybrid electrode.

The above XPD study was able to capture the timing of the reaction of lithium with CuS relative to its reaction with sulfur. However, the absence of new diffraction peaks related to copper sulfides indicates that the reaction products are not crystalline. *Operando* X-ray Absorption Spectroscopy on Cu has been conducted to provide more information. Figure 3A and B show a series of *operando* Cu K-edge XANES spectra recorded during discharge and charge half-cycles, respectively, of a sulfur-CuS hybrid electrode. The corresponding electrochemical curve is presented in Fig. 3C. No spectral changes could be observed in the XANES region between points *a*-*e*. In contrast, at point *f*, two changes in the XANES line shape emerge: first, feature I, which is characteristic for CuS^33^, in Fig. 3D becomes less prominent and the edge jump shifts to lower energies, as marked with feature II in Fig. 3D. Comparison of the *operando* spectra with those of the standards suggests that the spectral changes are consistent with depletion of sulfur, that is, the transition of CuS to Cu_1+x_S. The XANES spectra evolution is consistent with the XPD data indicating no phase transformation of CuS in the bulk sulfur-CuS hybrid electrode until the end of discharge.

To quantify the extent of the transition, the EXAFS region of the spectra were analyzed. The EXAFS spectra along with the fits (Figure S2) and the best-fit coordination numbers (CNs) and Cu-S interatomic distances are given in Table 1. It should be noted that the Cu-S phase diagram is extremely complex, with multiple stable and metastable stoichiometries^34^. In addition, Cu_2_S structure is intricate with 96 copper atoms in a monoclinic unit cell^35^, so only an average interatomic distance could be reliably established. After full discharge, the Cu-S CN is reduced from 2.5 to 2.1, with the corresponding elongation of the average bond length from 2.27A to 2.29A. Under the assumption that the values correlate directly with stoichiometry, the average composition of the discharged material will be approximately Cu_1.3_S. During charging, a very small increase in the intensity of feature I is observed (Figure S3), but there is no statistically significant change in the CN value, indicating that sulfur is not re-inserted into the material during charge. In order to understand whether the Cu_1+x_S phase is a single or composite phase, we attempted to fit the XANES spectra as a combination of principal components, represented by Gaussian shapes. For all standard components (CuS and Cu_2_S) and the sample discharged *operando*, the XANES spectra can be fit with three components, which suggest that in all cases the single chemical compound and not a mixture may be present in the sample (Figure S4)^36^. This implies that after the Li-S battery is fully discharged, the CuS was likely converted to a single phase with stoichiometry between CuS and Cu_2_S; however, further XAS experiments and simulation such as Density Functional Theory (DFT) calculations may be required to unequivocally determine the precise phase composition.

**Table 1:** Interatomic distance and Debye-Waller factors of sulfur-CuS hybrid electrode after discharge and standards.

Sample	Cu-S coordination number	Cu-S distance, Å	Debye-Waller factor
CuS (standard)	2.5 ± 0.2	2.272 ± 0.002	0.01± 0.001
Cu_2_S (standard)	1.5 ± 0.3	2.315 ± 0.02	0.02± 0.003
Discharged (point *f*)	2.1 ± 0.2	2.289 ± 0.02	0.01± 0.003

### Local elemental and chemical evolution by *operando* synchrotron x-ray fluorescence microscopy

**Figure 4:**
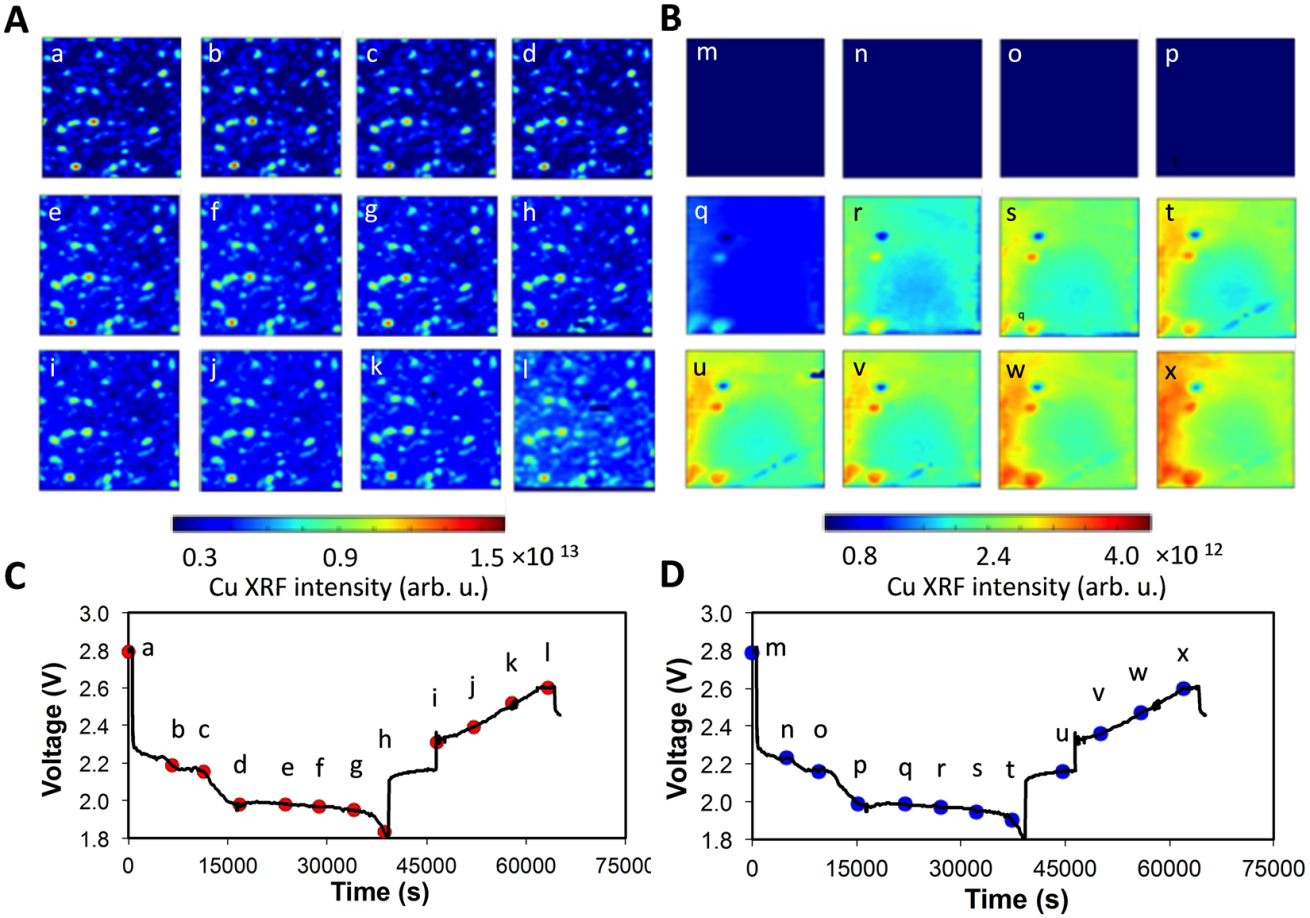
The *operando* XRF microscopy showing Cu distribution evolution: (**A**) for cathode and (**B**) for anode. (**C**) and (**D**) both show the capacity-voltage plot of the sulfur-CuS hybrid cathode cell, each data point in a)-x) are labelled in (**C**) for cathode and (**D**) for anode; the field of view is 160 µm × 160 µm in size.

The above *operando* XPD and XAS provided further mechanistic understanding of the sulfur-CuS hybrid cathode cell under cycling. To visualize the CuS dissolution phenomena and pin-point the onset of the dissolution from the sulfur-CuS hybrid cathode and its redeposition at the Li anode, we conducted XRF *operando* experiments. The XRF microscopy provides the ability to image the elemental distribution evolution, which can then be correlated with the bulk chemical and structural evolution as measured by XPD and XAS. Figure 4A and B show Cu XRF images collected from the cathode and the anode in a sulfur-CuS hybrid electrode at different levels of lithiation during a full lithiation/de-lithiation cycle. Each mapping graph is correlated with an individual data point indicated in the voltage profile in Fig. 4C (cathode) and Fig. 4D (anode). Figure 4A and B show that CuS particles or agglomerates are in the form of discrete clusters. Therefore, each CuS particle was estimated to be 10–20 μm in size. It can be seen that in Fig. 4A point a-d, which corresponds to the 1^st^ sloped discharge region of sulfur electrode, no evident change was observed on the CuS particles. However, when the electrochemical reactions proceed shortly beyond the sloped region, a sudden blurriness in the images starts to develop on each of the CuS particles in the field of view and Cu fluorescence intensity also starts to increase, as shown in Fig. 4A point e. In addition, no copper XRF signal was observed on the lithium anode surface untill Fig. 4B point p, which also corresponds to the end of the sloped discharge region of sulfur-CuS electrode. However, immediately after the discharge entered into the plateau region of sulfur component, a strong copper signature starts to develop in Fig. 4B point q. The coincidence of the onset of copper deposition on the lithium anode and the morphological change of the CuS particles in the cathode suggest that the latter is not simply the copper redistribution or volume change after the lithiation of CuS, but the initiation of copper ion dissolution in the electrolyte. The overall increase of copper XRF signal can be explained by some copper ions diffused from a deeper region of the electrode towards the surface or into the electrolyte, contributing to higher XRF signals. It has been already demonstrated in the *operando* XPD study that CuS does not start lithiation until the end of the whole discharge process. Interestingly, the onset of copper ion dissolution almost exactly coincides with the start of the plateau discharge region of sulfur. In the original study, it was determined that the species responsible for the dissolution of CuS is developed elsewhere during the lower-voltage plateau discharge of sulfur by performing *ex situ* characterizations^25^. Here, with the help of *operando* observation, the original hypothesis was validated and further refined - the species develops as soon as the plateau-region discharge of sulfur begins. Based on this, the species seems also to be a signature of the low-voltage plateau region of sulfur discharge. It cannot be generated by disproportionation of higher order polysulfides S_n_^2−^ (4 ≤ n ≤ 8) commonly observed in the sloped discharge region^5^. Recent *operando* studies of polysulfide evolution during sulfur electrode discharge has also provided enough evidence that S_3_^2−^ is generated in the initial sloped discharge region^37–39^, so S_3_^2−^ cannot be associated with the sloped-plateau transition point at 21% of cell discharge either. Therefore, the species which mostly likely lead to the dissolution of CuS are S_2_^2−^ and S^2−^.

**Figure 5:**
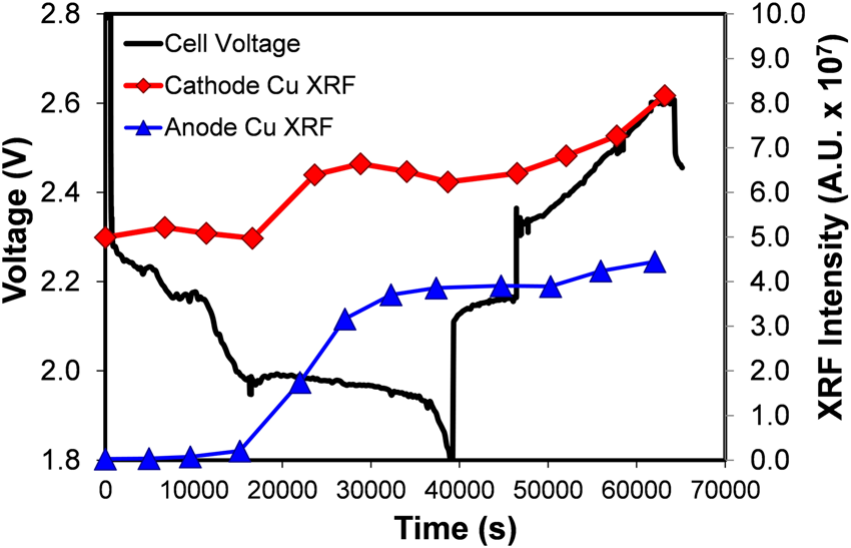
Integrated XRF intensity on cathode (red) and anode (blue) plotted together with capacity-voltage profile of sulfur-CuS hybrid electrode.

While the Cu distribution maps in Fig. 4 directly visualize the onset of the Cu ion dissolution, quantitating the changes is more difficult. In Fig. 5, we show the total Cu fluorescence, obtained by integrating over the field of view for each time point in Fig. 4A and B. The Cu fluorescence intensity of each frame in Fig. 4 is plotted versus time in Fig. 5 for cathode and anode. On the cathode side, the intensity integration is constant at ~5 × 10^7^ until 16611 seconds, and starts to increase after this time point. This corresponds to Fig. 4A point e, where the cathode Cu XRF map starts to become blurrier as Cu ion dissolution begins. Again, the total intensity increase is most likely due to the out-diffusion of dissolved Cu ions from the CuS particles embedded in the electrode. The Cu fluorescence intensity increases until 28828 seconds and then levels off until 38698 seconds, which is near the end of the discharge; little change was observed during the open-circuit period. The quantitative treatment of fluorescence intensity also helps to further infer that S^2−^ alone could not be the species that enhances the dissolution of Cu ions. Crystalline Li_2_S was not observed until 43% cell discharge, or close to the middle of the plateau discharge region, in the XPD spectrum of Fig. 2B. This means that S^2−^ is in an over-concentrated state versus that determined by the solubility of Li_2_S at this point and until the end of the Li_2_S deposition, which is at around 784 mAh g^−1^ (85% of cell discharge) in Fig. 2B, as determined from Li_2_S (111) peak intensity evolution. However, the intensity of Cu at the cathode starts to drop at 28828 seconds and the slope of increasing Cu intensity at the anode side starts to decline at 27060 seconds in Fig. 5, the former time point corresponding to 74% and the latter to 70% of cell discharge. Both of these transitions are signs of the slowing-down of Cu dissolution, while S^2−^ is still in an over-concentrated state and in large excess for Li_2_S deposition based on XPD analysis. Based on this, it is inferred that S^2−^ does not play a vital role in the slope to plateau discharge transition of sulfur electrode and Cu dissolution in CuS component. Therefore, S_2_^2−^ seems to be the most probable species involved in both processes since the other candidates are excluded. In the future, strategies to reduce or eliminate this interaction can be studied to mitigate such parasitic reactions.

In Fig. 5, after the recharge process starts at 46493 seconds, the Cu fluorescence intensity again starts to rise to even higher levels and continues until the end of the charge process. In addition, the total intensity change is ~1.7 × 10^7^ AU, similar to the change 1.6 × 10^7^ AU observed in discharge from 16,611 seconds to 28,828 seconds. This observation indicates the possibility of another copper ion dissolution step during the cell charging. The trend observed on the anode side is also in agreement with the conclusions draw from the cathode data, as shown in Fig. 5. Although this explanation is still consistent with our previously reported observation^25^, additional follow-up studies are necessary to fully understand this phenomenon. Notably the metal ion dissolution during sulfur cell charging has also been observed in the sulfur-FeS_2_ hybrid system^40^, in which Fe-ion dissolution happened only during recharging the fully discharged cell.

### Mechanistic understanding by synchrotron multi-modal approach

**Figure 6:**
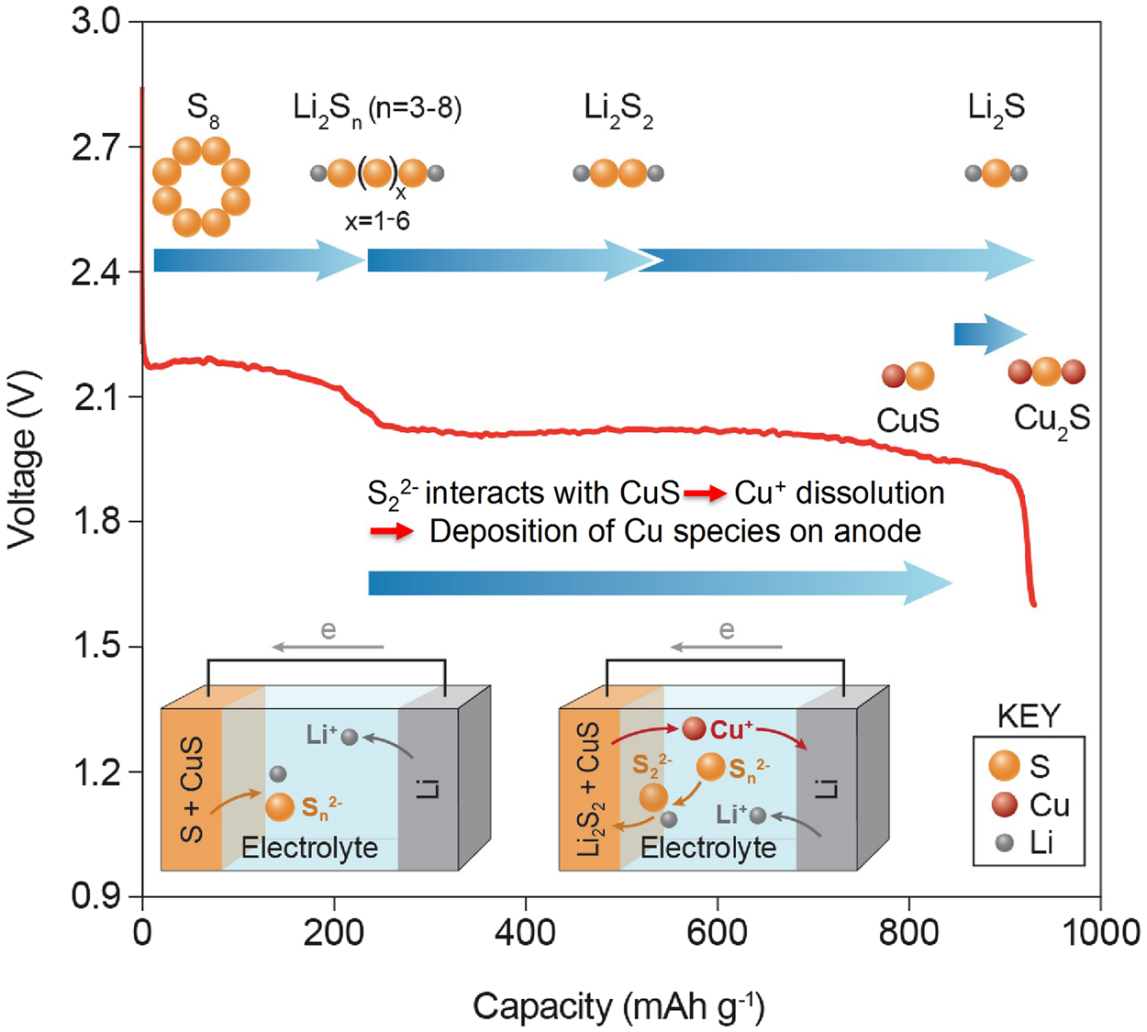
Mechanism of sulfur-CuS hybrid cathode cell discharge.

Taking into account of all the complementary information from the multi-modal synchrotron x-ray experiments including *operando* XPD, XAS and XRF, the evolution of the sulfur-CuS hybrid electrode material crystalline phase and the mechanism of CuS dissolution during cell discharge are summarized in Fig. 6. The crystalline sulfur cathode is completely consumed during the initial 21% of cell discharge, presumably converted to the electrolyte-soluble high-order polysulfide (Li_2_S_n_, where n = 3 to 8). Starting from ~25% to ~43% cell discharge, or the beginning of the plateau discharge region, the polysulfide is converted to amorphous phase Li_2_S_2_, followed by conversion of Li_2_S_2_ into Li_2_S by additional lithiation up to ~85% cell discharge. The lithiation of CuS starts at the very end of discharge to form amorphous Cu_1+x_S at ~1.95 V. During this process, CuS interacts strongly with soluble low-order polysulfide species, most probably with S_2_^2−^ at the beginning of the 2.0 V plateau region. The dissolved Cu ions then migrate from the cathode side to the anode side through the electrolyte. Reduction and deposition of copper species on the anode surface altered the anode SEI, causing the fast capacity-fade as observed in the previous report^25^. This work provides further mechanistic insight regarding the interaction between sulfur and CuS in Li-S battery.

## Methods

### *Operando* battery cell preparation

Battery cells were prepared for XPD, XAS and XRF *operando* experiments; one cell design was optimized to enable measurements with all three techniques at different synchrotron beamlines. The design enables several important features, which are described in the result and discussion section. A center polypropylene tube was used to construct the battery, with a total diameter of 2.92 mm and a wall thickness of 310 µm. The current collectors are aluminum rod, for the cathode, and stainless steel rod, for the anode. Sulfur-CuS hybrid cathode slurry was directly coated onto the surface of the aluminum rod and the anode is a small lithium cube ~1 mm^3^ in size. During assembly, a layer of separator (Celgard 2325) was sandwiched between the cathode and anode to prevent shorting. The cell was then sealed by epoxy sealant. The cathode slurry in this study is composed of sulfur:CuS:Super C65:PVDF = 45:15:30:10 by weight. Super C65 carbon black is obtained from Imerys and the other components are all from Alfa Aesar. The ingredients were mixed in N-Methyl-2-pyrrolidone (NMP, Sigma Aldrich) and coated onto the top of the aluminum rod. It was dried under continuous dry airflow for 24 hours at 50 °C before use in a dry room with dew point < 40 °C. The electrolyte used for testing was LITFSI (1.0 M) dissolved in 1,2-dimethoxyethane (DME) and 1,3-dioxolane (DOL) (1:1 ratio, by volume) obtained from BASF. 1wt.% of LiNO_3_ is added to enhance coulombic efficiency. The total electrolyte was estimated to be 20 µL.

### Synchrotron x-ray experiments

Synchrotron XPD, XAS and XRF experiments were performed at different beamlines at National Synchrotron Light Source - II (NSLS-II) of Brookhaven National Laboratory. The *operando* XPD experiment was conducted at the X-ray Powder Diffraction beamline (XPD, 28-ID-2)^41^. A large-area amorphous-silicon digital X-ray detector with 2048 x 2048 pixels was used to collect the diffraction patterns with size of each pixel 200 × 200 microns. The sample to detector distance was first calibrated by using a Ni standard and determined to be 1497.768 mm. The x-ray wavelength was 0.183775 Å (x-ray energy was 67.465 keV) and beam size was 0.5 mm × 0.5 mm. During the XPD data collection, the Li-S battery with a sulfur-CuS hybrid electrode was discharged at a C/22 rate down to 1.8 V. The whole discharge process took about 682 minutes. The *operando* XPD experiment was conducted by collecting a two dimansional (2D) XPD pattern every 15 minutes during the battery discharge. The exposure time was 20 seconds per 2D image. XPD patterns were also collected on the standard samples CuS and Cu_2_S. The *operando* XAS experiment was conducted at the Inner-Shell Spectroscopy beamline (ISS, 8-ID)^42^. The ISS beamline uses a damping wiggler source which provides a high photon flux (~ 5 × 10^13^ ph/s) and has an energy range from 4.9 keV to 36 keV. A 0.8 mm × 0.3 mm (h × v) spot size was used to illuminate the sample, controlled by a set of slits. Cu K-edge absorption spectra were acquired using a cryogenically cooled double crystal Si (1111) monochromator in a fly-scan mode. Spectra were recorded in the fluorescence mode with a passivated implanted planar silicon (PIPS) detector. Scanning and data acquisition time of two spectra on one sample was ~ 1 min. XAS spectra were collected from the sulfur-CuS hybrid cathode every 8 minutes during the *operando* ISS experiment, where the Li-S battery was discharged/charged under C/20 rate. Absorption spectra of CuS and Cu_2_S (Alfa Aesar) standards were prepared into a disk pellet using boron nitride as a matrix and the spectra were taken in transmission mode. The *operando* XRF Microscopy experiment was conducted at the Sub-micron Resolution X-ray Spectroscopy beamline (SRX, 5-ID)^43^. The incident x-ray energy was 10 keV, focused onto the sample by a set of Kirkpatrick-Baez mirrors with a spot size ~ 1 micron. XRF experiment was conducted by raster scanning the sample against the incident x-ray beam, and the full XRF spectrum from the sample at each scanned point (pixel) was collected by a silicon drift detector. During the XRF experiment, the sulfur-CuS hybrid electrode was discharged and charged at a C/32 rate. The *operando* XRF experiment was conducted by collecting XRF images from both the cathode and anode sides of the battery cell consecutively, with each 160 x 160 microns image taking ~25 min. The C-rates used in this study for different measurements were chosen to ensure that a sufficient number of data points were collected during the electrochemical discharge/charge of the batteries, given the the timing requirements of different beamlines and techniques used in this study. All of the C-rates used here result in a sufficiently slow cycling rate and, thus, the data remain representative for the reactions.

### Data Analysis

Each XPD pattern was integrated azimuthally and reduced to an intensity vs. 2θ (diffraction angle) plot. The peak locations were then compared with the standard references to identify the corresponding phases using commercial software package Jade (Materials Data, Inc.). The standard patterns collected during the experiment were compared with the references in the database and found to be consistent. Li_2_S (111) is the only peak that has sufficient intensity for Li_2_S in our experiment. It is also the strongest peak according to the database. Because the Li_2_S (111) peak overlaps with S (311) and CuS (100) peaks, the intensity of Li_2_S (111) could not be measured directly. However, the changes in the diffraction peak area integral of S (311) and CuS (100) relative to the pristine sample can be calculated based on the changes in individual peaks such as S (222) and CuS (103) relative to the pristine sample. This is because the diffraction peak area integral of a specific phase is proportional to its weight fraction, and also there is no preferred crystalline orientation (texture) observed in the sample based on the 2D diffraction pattern. Li_2_S (111) peak area can then be calculated by subtracting the sulfur and CuS contribution from the total integrated area in 2θ range from 3.14134° to 3.22656°. Other diffraction peaks were not entered in this analysis since their intensities were lower, but the relative variations of the intensities were found to be consistent across all detectable diffraction peaks. The Athena software^44^ was used to process the XAS data. Normalization and background subtraction of the XAS spectra were conducted in Athena: a line is used to fit pre-edge region and a quadratic polynomial is regressed to the post-edge region. The Artemis software with FEEF was used to conduct modeling in the EXAFS part of the data based on theory well-described elsewhere^44^. The modeling provides the Cu-S coordination number and neighboring radial distance. The data fitting of the XRF spectra was accomplished using PyXRF software^45^ to quantify the Cu XRF signal in each of the scanning XRF images.

## Electronic supplementary material

Supporting Information
