# Supplementary material for: Operando Multi-modal Synchrotron Investigation for Structural and Chemical Evolution of Cupric Sulfide (CuS) Additive in Li-S battery
Source: Sci Rep. 2017 Oct 11;7:12976. doi: 10.1038/s41598-017-12738-0 (PMC5636834; doi:10.1038/s41598-017-12738-0)
Supplement: Supplementary file 1 — Supporting Information [file 41598_2017_12738_MOESM1_ESM.pdf]

Supporting Information

Operando Multi-modal Synchrotron Investigation for Structural and Chemical Evolution of Cupric Sulfide (CuS) Additive in Li-S battery

Ke Sun[1‡], Chonghang Zhao[2‡], Cheng-Hung Lin[2‡], Eli Stavitski[3], Garth J Williams[3], Jianming Bai[3], Eric Dooryhee[3], Klaus Attenkofer[3], Juergen Thieme[3], Yu-chen Karen Chen-Wiegart[2,3*], Hong Gan[1*]

[1] Sustainable Energy Technologies Department, Brookhaven National Laboratory, Upton, NY 11973
[2] Department of Materials Science and Chemical Engineering, Stony Brook University, Stony Brook, NY 11794
[3] National Synchrotron Light Source II, Brookhaven National Laboratory, Upton, NY 11973

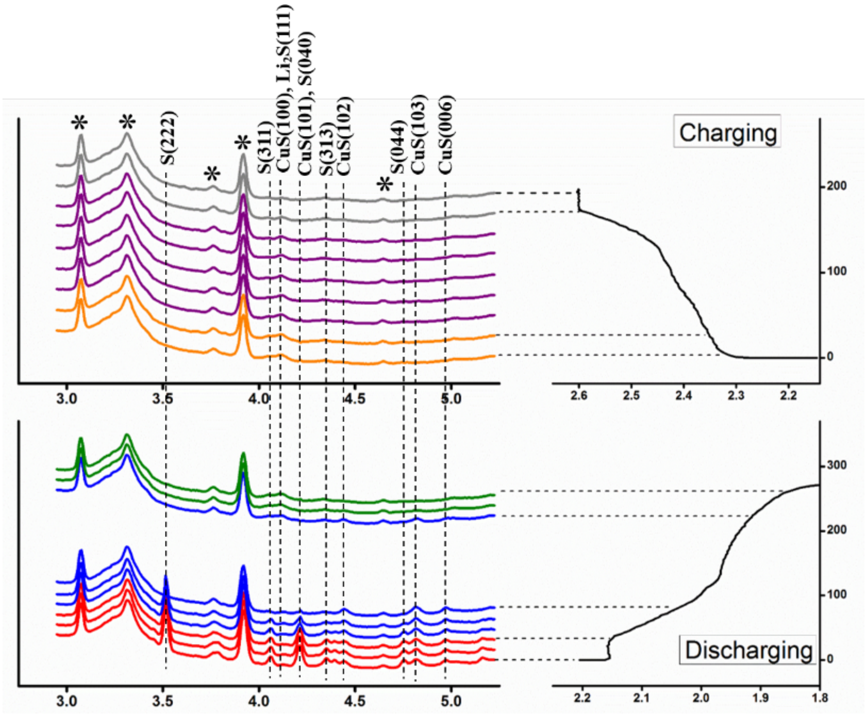

Figure S1: Operando XPD patterns of S-CuS hydrid electrode collected during discharging and charging, aligned with capacity-voltage profile of sulfur-CuS electrode; diffraction peaks from the tube are labelled with "*".

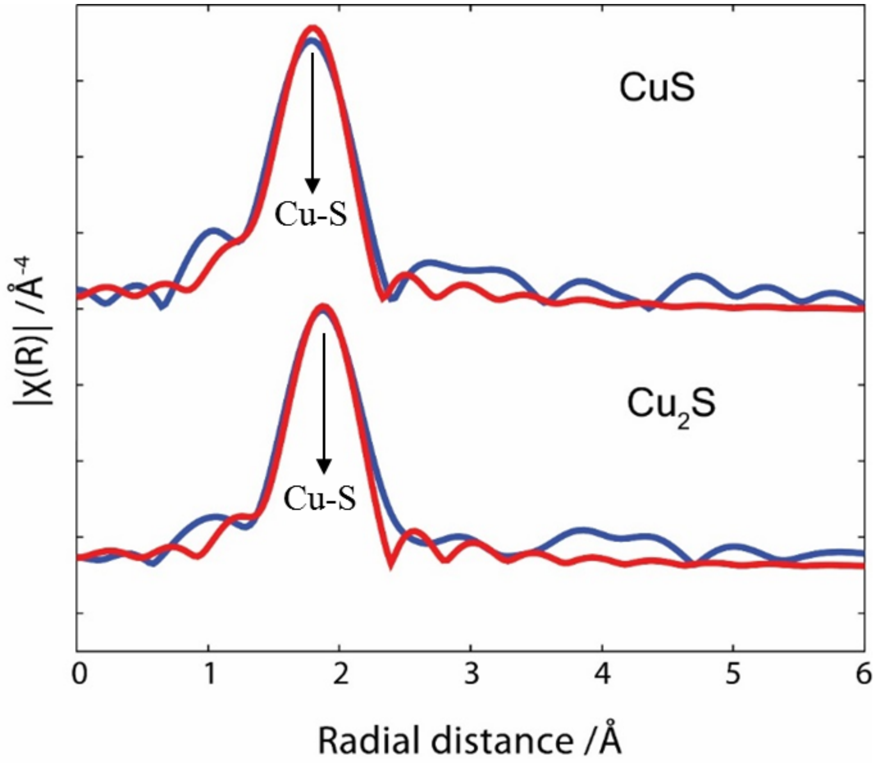

Figure S2: The radial distribution function of CuS and Cu_2S standard materials derived from EXAFS Fourier transform (blue) and fits (red) using the parameters given in Table 1. Arrows indicate the copper-sulfur (Cu-S) coordinate.

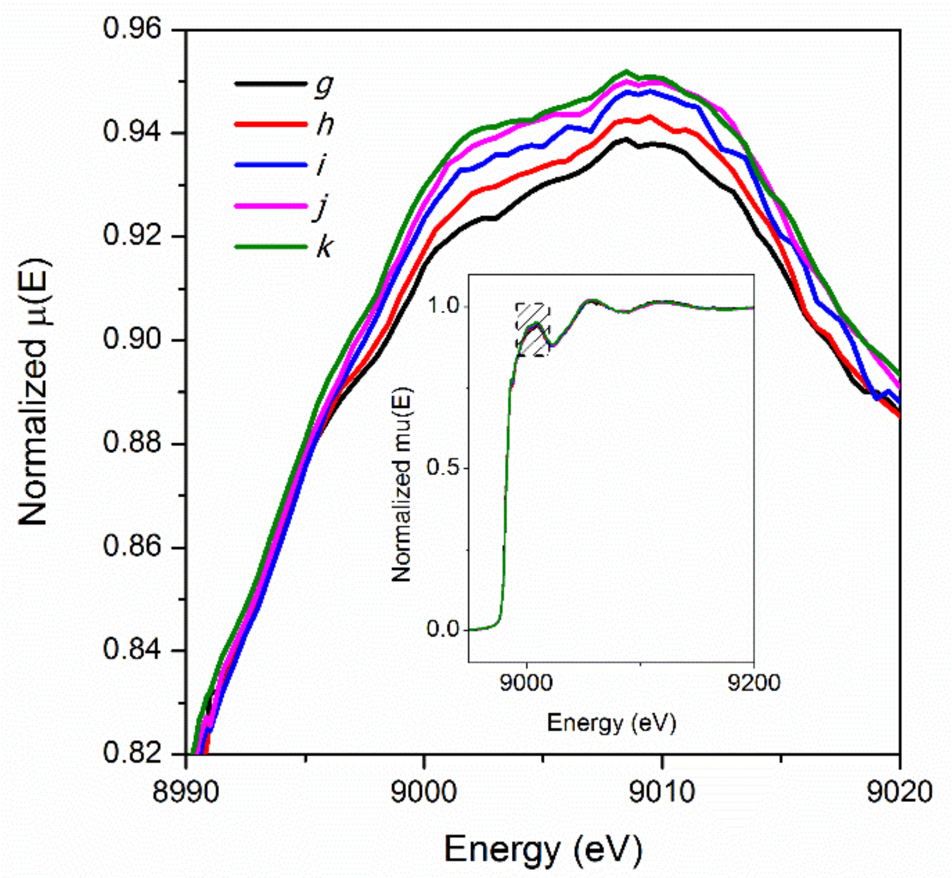

Figure S3: The magnified view of selected area (as displayed in the insert figure) from XANES spectra at charging region, corresponding to points g-k in Figure 3B & C.

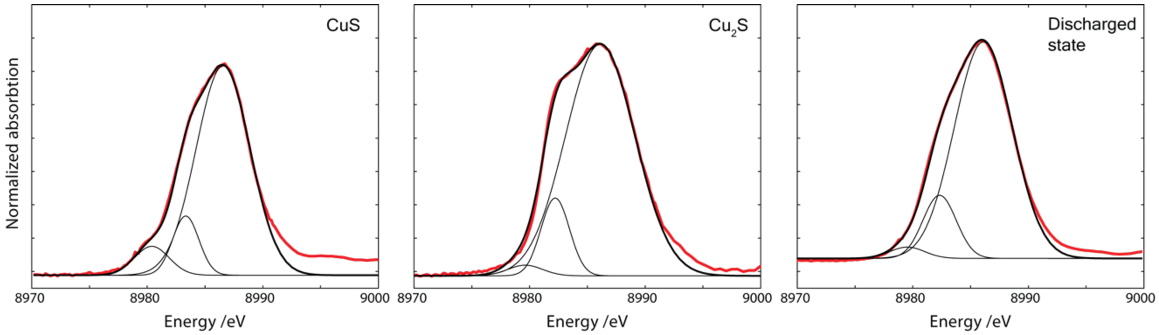

Figure S4: Fitting of XANES region of the XAS spectra for the standard materials and fully discharged state. The spectra, after substracting the edge represented by the arctangent function, are modelled with three Gaussian profiles. These features realted to core-to-valence electronic transitions in Cu_xS_y materials are described in Legros et al., 2010, section: XANES Cu Speciation on the Molecular Scale, Investigation of Copper Speciation in Pig Slurry by a Multitechnique Approach; further elucidation of the XANES spectral changes as a function of charge state with be a subject of future publications.
